# Account of Diseases in an Eastern District of London, from November 20, to December 20, 1803

**Published:** 1804-01-01

**Authors:** 


### *Account of Diseases in an Eastern District of London, from November 20, to December 20, 1803.*

| | |
|---|---|
| ACUTE DISEASES. | |
| Pneumonia | 3 |
| Peripneumonia Notha | 7 |
| Dysenteria | 5 |
| Variolæ | 5 |
| Rheumatismus Acutus | 4 |
| CHRONIC DISEASES. | |
| Tussis | 19 |
| Tussis cum Dyspnœa | 17 |
| Hydrothorax | 3 |
| Phthisis Pulmonalis | 4 |
| Hepatitis Chronica | 2 |
| Ascites | 3 |
| Anasarca | 5 |
| Diarrhœa | 9 |
| Enterodynia | 7 |
| Gastrodynia | 5 |
| Menorrhagia | 7 |
| Amenorrhœa | 12 |
| Leucorrhœa | 10 |
| Dysuria | 4 |
| Hysteria | 3 |
| Cephalalgia | 8 |
| Paralysis | 3 |
| Vertigo | 2 |
| Rheumatismus Chronicus | 20 |
| PUERPERAL DISEASES. | |
| Menorrhagia Lochialis | 7 |
| Ephemera | 8 |
| Dolores post Partum | 4 |
| INFANTILE DISEASES. | |
| Ophthalmia Purulenta | 2 |
| Herpes | 3 |
| Vermes | 2 |
| Dentitio | 4 |

The state of diseases has of late been more favourable ~~than usual~~. Fewer of those complaints have appeared which in general occur in the autumnal months. Coughs and catarrhs, difficult respiration, and other affections of the chest have indeed increased in number, and have been attended with an aggravation of symptoms since the late alteration in the state of the atmosphere.

The subject which engages the principal attention of medical men at present, is the state of variolous contagion. The small-pox has lately appeared in a much larger number of instances than has been observed for a considerable time. This fact is very well ascertained by the report of the weekly bills of mortality, in which the number of deaths occasioned by this disease is very considerable; and also by the observation of medical men, who in the course of their practice have met with an unusual number of cases. This disease has not only occurred very frequently, but has been attended with aggravated symptoms, and has often proved fatal. That the small-pox should prevail in so considerable a degree, when such means are employing for the prevention of it, may excite some surprise. It must be recollected, however, that notwithstanding the very extensive trial which has been made of the Vaccine Inoculation in every part of our own country, and in other parts of the globe, globe, and the uniform success which has attended it, there is still a considerable prejudice against it. That requests are frequently made for inoculation with variolous matter, is known by private practitioners of medicine, and especially by those whose connection with public institutions gives them a still farther opportunity of observing the fact.

How desirable the removal of this prejudice is must appear to every reflecting person, to whom it must occur that by the preference of the variolous to the vaccine inoculation, the former disease is propagated, and that the public are in continual danger of suffering by a malady which has proved one of the most loathsome and dangerous of those that have ever visited the human frame.

